# Pathogenic *Leptospira* spp. Seroprevalence and Herd-Level Risk Factors Associated with Chilean Dairy Cattle

**DOI:** 10.3390/ani11113148

**Published:** 2021-11-04

**Authors:** Victor Montes, Gustavo Monti

**Affiliations:** 1 PhD Program, Faculty of Veterinary Sciences, Universidad Austral de Chile, Valdivia 5090000, Chile; mon_vet77@yahoo.es; 2 Department of Veterinary, Faculty of Veterinary Sciences, Universidad Técnica de Manabí, Portoviejo 130701, Ecuador; 3 Department of Preventive Veterinary Medicine, Faculty of Veterinary Sciences, Universidad Austral de Chile, Valdivia 5090000, Chile; 4 Quantitative Veterinary Epidemiology Group, Wageningen University and Research, 6708 PB Wageningen, The Netherlands

**Keywords:** *Leptospira*, within-herd seroprevalence, herd-level prevalence, risk factors, dairy herds

## Abstract

**Simple Summary:**

Leptospirosis is a ubiquitous distributed infectious disease present in wild and domestic animals that can be transmitted to humans. This study aimed to estimate the burden of the bacteria in dairy cows from southern Chile and identify the factors associated with the herd-level status. We studied 147 herds and 4876 lactating cows from the area, and an infected herd was defined when at least one serologically positive reactor to MAT was detected. An epidemiological survey was applied to the herd’s owner. The estimated overall individual prevalence was 5.3% (95% CI 2.9–7.7), the overall herd-level prevalence was 42.2% (95% CI 34.2–50.2), and there was variation in both between different herd sizes. *L. borgpetersenii* serovars Hardjo and Tarassovi and *L. interrogans* serovar Pomona were the more frequent serovars in *Leptospira* non-vaccinated herds. Attenuated *Leptospira* vaccine usage was assessed as a factor that decreases the risk of a farm being infected (OR = 0.04; 95% CI = 0.02–0.11), and variables that increase that risk were using bulls for mating (OR = 3.43; (95% CI = 1.1–10.1) and continuous calving distribution (OR = 3.4; 95% CI = 1.3–8.8). The results from this study will contribute to unravelling the infection burden in the main dairy area of the country and designing control strategies.

**Abstract:**

Leptospirosis is a ubiquitous distributed zoonotic infectious disease present in wild and domestic animals. This study aimed to estimate within-herd and herd-level seroprevalence against pathogenic *Leptospira* spp. in dairy cows from southern Chile and identify risk factors associated with the herd-level status. We used a multi-stage strategy combined with a stratified sample strategy for randomly sampling 147 herds and 4876 lactating cows. We considered as infected a herd with at least one positive reactor to MAT. In addition, an epidemiological survey was applied to the herd’s owners and a logistic regression (LR) model was constructed to analyze it. The overall within-herd prevalence was 5.9% (95% CI 4.9–6.8), the overall herd-level prevalence was 42.2% (95% CI 9.2–47.9), and there was variation in both between different herd sizes. *L.* *borgpetersenii* serovars Hardjo and Tarassovi and *L. interrogans* serovar Pomona were the more frequent serovars in non-vaccinated herds. A factor that decreases the risk of a farm being infected was *Leptospira* vaccine usage (OR = 0.04; 95% CI = 0.02–0.11), and variables that increase risk were using bulls for mating (OR = 3.43; 95% CI = 1.1–10.1) and continuous calving distribution (OR = 3.4; 95% CI = 1.3–8.8). The study’s results will contribute to unravelling the infection burden in the main dairy area of the country and designing control strategies.

## 1. Introduction

Leptospirosis is a globally distributed zoonotic infectious disease that is especially frequent in tropical areas. However, in temperate countries, human cases also occur, though this is frequently incidental, due to travelling abroad to tropical countries or from exposure related to recreational activities [1]. In addition, Leptospirosis has been identified as a re-emerging zoonotic disease affected by global climate change [2,3].

Transmission of pathogenic *Leptospira* spp. is possible either through direct contact with infected carrier animals or indirectly through contaminated sources, such as water. One relevant source of human infection is rodents [4], but many other wild and domestic animals can be reservoir hosts and shed leptospires [5]. Cattle are one of these species and can be a source of infection and infected cattle could suffer reproductive failure, abortion, stillbirths, fetal mummification, and producing weak calves [6]. Urine from infected cattle can be an infection source for humans, but transmission from cattle to humans is also possible through aborted fetuses or vaginal discharges after abortion or calving [5]; therefore, cattle can pose a possible threat to the health of their owners or persons they are in contact with, such as veterinarians and milkers [7,8,9,10].

Serological testing is the most widely used method for diagnosing at herd level, and MAT is the standard serological test [5]. Several serological prevalence studies in cattle at individual or herd level are described, especially in areas where the presentation of Leptospirosis is high. For example, studies estimating individual seroprevalence in Brazilian cattle have reported a seroprevalence of 35.9% to 61.1% [11,12,13,14] and a herd-level prevalence between 64.8% and 89.7% [11,12,13,15]. In Madagascar, an overall seroprevalence of 59.3% (95% CI = 52.0–66.2) was estimated; and in Jordan, an individual seroprevalence of 27.0% and an overall seroprevalence of 92% was estimated [16]. In contrast, in temperate countries, such as New Zealand, individual seroprevalence for beef cattle was estimated to be 45.6% (95% CI = 43.3–47.9) for *L. borgpetersenii* serovar Hardjo and 19.6% (95% CI = 17.9–21.5) for *L. interrogans* serovar Pomona; herd-level prevalence for Hardjo varied from 79.0% to 90.5% and for Pomona from 42.1% to 68.4% [17]. In Europe, in Spain, a within-herd seroprevalence of 8% and a herd-level seroprevalence of 42.2% was reported [18] and in Ireland, a within-herd seroprevalence of 65.7% and a herd-level seroprevalence of 91% was reported [19].

Although in Chile, it has been a notifiable disease in humans since 2001, studies on leptospirosis are scarce [20,21]; it is suspected to be underreported [22], as in many countries. In addition, it is an occupational disease associated with people in contact with animals and poor rural communities [23].

The Chilean cattle stock is approximately 4 million and produces around 2321 million liters of milk per year. Los Lagos and Los Rios regions represent 45% of this cattle stock and 76% of the milk production, becoming important regions of meat and milk production in the country [24]. Nevertheless, high abortion rates in these provinces are reported [25], and Leptospirosis is endemic in the country [26].

Chile has reported few, outdated, studies on within-herd or herd-level seroprevalence [21], and the most recently reported study estimated a within-herd seroprevalence ranging from 2% to 75%, with a median of 15%, and a herd-level prevalence of 75%; however, the study targeted only small farmers [26], nevertheless demonstrating that this infectious agent is present in the environment.

Bovine leptospirosis occurs worldwide and results from infection by a wide variety of serovars [5]. However, the most frequently reported is the *L. borgpetersenii* serovar Hardjo (Hardjobovis, HB), but *L. interrogans* serovar Hardjo (Hardjoprajitno, HP) also occurs in cattle in some parts of the world [5]. In Chile, both are present [26,27,28] in cattle populations.

In the literature, several risk factors have been associated with exposure to pathogenic *Leptospira* spp. in dairy cattle, such as environmental conditions, management systems, and herd characteristics [12,13,16,17,29,30]. In addition, co-grazing with other animals, such as equines, sheep, goats, pigs, deer, or capybaras [13,17,29,30], and grazing in flooded pastures are considered as risk factors [12].

This study aimed to estimate within-herd and herd-level seroprevalence against pathogenic *Leptospira* spp. in dairy cows from southern Chile and identify risk factors associated with the herd-level status. The results from this study will contribute to unravelling the infection burden in the main dairy area of the country.

## 2. Materials and Methods

### 2.1. Study Population, Sample Size Estimation, and Selection of Herds

The study population corresponded to dairy herds located in southern Chile in Los Rios and Los Lagos regions, which are the main dairy production area in the country, holding 44.5% of the Chilean cattle population [31].

This study was part of a large project that included several pathogens (bovine viral diarrhea virus and *Mycobacterium avium subsp. paratuberculosis*). A cross-sectional study was performed using a complex survey strategy for accounting for the hierarchical structure of the population (two-stage cluster combined with stratified sampling). Dairy farms were randomly selected from the sampling frame, the Animal Health Services database. The selection was stratified by herd size, defined as small (<100 cows), medium (100–200 cows), and large (>200 cows). The percentage of herds in the sample was proportional to the regional population size (65%, 25%, and 10%, respectively). The eligible population were 150 dairy herds that were randomly selected from Los Ríos and Los Lagos regions. The source population for this study was the 10,859 dairy herds present in both regions [32]; the expected herd prevalence was 50% (for more conservative sample size), with an accepted error of 8% and 95% confidence. The sample was weighted to account for differential sampling probabilities and represent the distribution of the different herd sizes in the region based on [32].

Between July 2011 and August 2012, each herd enrolled in the study was visited by the project’s team, and during each visit, all lactating cows were sampled. However, given resource limitations, a random and representative sample of them for each herd was tested and later analyzed, instead of all of them being processed. To estimate the number of samples to be processed for each herd, we considered a simple random sample of a finite population (represented by the number of lactating cows in the herd), with an expected within-herd prevalence of 50% (the most conservative one for testing proportions), an accepted error of 8%, and a 95% confidence level. Once we estimated the number of samples to be tested, we selected the cows by a random number generator.

During the visits, 12,311 blood samples were obtained from lactating cows and of these, 4998 samples were finally selected and processed. For estimation of the prevalence of pathogenic *Leptospira* spp. at the herd level, a herd was considered infected at the herd level if at least one animal with a positive result to microscopic agglutination test (MAT) was found.

Manipulations performed on animals were approved by the Universidad Austral Animal Ethics Committee, protocol 15/2010.

### 2.2. Field and Laboratory Procedures

Five mL of blood was obtained with vacutainers from the coccygeal vein after disinfection of the area. The samples were kept cold until they were received at the Microbiology laboratory, Universidad Austral de Chile; serum was obtained, and then samples were stored at −86 °C until processing.

Serum samples were tested against nine serovars. Panel 1 included the following serovars: *L. interrogans* serovars Pomona, Canicola, Icterohaemorrhagiae, and Autumnalis and *L. borgpetersenii* serovars Ballum and Hardjo, which correspond to the most frequent serovars present in South Chile [26]. In addition, another three serovars (*L. interrogans* serovar Bratislava, *L. kirschneri* serovar Grippotyphosa, and *L. borgpetersenii* serovar Tarassovi) were included in panel 2 to broaden the former. MAT was performed as described by Salgado et al. [26], based on the method described by [33]. Nine live antigens were grown in the liquid medium Ellinghausen–McCullough–Johnson–Harris (EMJH), and a standardized concentration of 2 × 10^8^ bacteria was used. A positive result was considered when the sample showed 50% of agglutination for the serovar analyzed.

Currently, in Chile, commercial bacterin vaccines are available that use either a monovalent vaccine with *L. borgpetersenii* serovariedad Hardjo (Hardjo bovis) (Fortress^®^, Zoetis, NJ, USA), or a pentavalent vaccine with *L. interrogans* serovars Pomona, Canicola, and Icterohaemorrhagiae; *L. kirschneri* serovar Grippotyphosa; and *L. interrogans* serovar Hardjo (Hardjoprajitno) (Cattle master^®^ 4 + L5 or Leptoferm5, Zoetis, NJ, USA).

For all serovars, a reciprocal titer of ≥1:200 was considered positive for animals coming from non-vaccinated herds and ≥1:800 for vaccinated herds based on two studies that compared antibody responses after vaccination [34,35]. When a sample reacted to more than one serovar, the highest titer was specified as the cause of infection. However, reactions to different serovars at the same titer were considered co-agglutinations. Therefore, an animal was considered as pathogenic *Leptospira* spp. exposed when showing at least one positive result in any of the serovars included in both panels.

### 2.3. Data Analysis

Fisher’s exact test was used to compare proportions between regions of origin, herd size, history, and vaccine use.

Apparent herd prevalence (AHP) was estimated as the number of herds with at least one animal with a MAT-positive result divided by the number of total herds. The apparent individual prevalence (AIP) was estimated using several functions of the package “survey” (V.3.33-2) [36] of the software R (V3.6.3) [37].

### 2.4. Risk Factors for Pathogenic Leptospira spp. Herd Status

A questionnaire (Supplementary Materials) was administered in the enrolled farms to the owner or manager of a farm, followed by a personal interview by a trained veterinarian during blood sampling, to evaluate management practices and herd characteristics associated with the *Leptospira* herd-infection status under Chilean-pasture-based production and management conditions. The questionnaire was undeclared and pre-tested for reliability and validity in 10 dairy farms of the area before being applied, checked for question variation, meaning, task difficulty, and respondent interest and attention by the interviewed.

The questionnaire collected data on *Leptospira* vaccination and deworming protocols, feeding management, semen source, history of abortion, repeat breeders, water source, and availability of veterinary services. Additional details collected included the average size of the milking herd, the number of animals in each age class (calves, heifers, and adult milking cows), the predominant breed of animals in the milking herd, management practices for the different age groups, details of the presence of rats and other domestic animals resident on the property, the presence or absence of biosecurity practices, and whether or not cases of leptospirosis had been diagnosed in cows in the past.

Assessments of the association between potential risk factors and the pathogenic *Leptospira* spp. herd status were performed using a conditional logistic regression model. The variables were first selected using unconditional logistic regression models with each variable (*p* < 0.25), a conditional model was then constructed using a forward strategy for variable inclusion, and Bayesian´s Information Criteria (BIC) were finally used for assessing the goodness-of-fit of the different models. Odds ratios (OR) and their 95% confidence intervals (CI) for the variables included in the final model were estimated, and a *p*-value of <0.05 was used for assigning statistical significance. Additionally, interactions between the variables were evaluated on the basis of biological plausibility and potential confounders. All of the statistical analyses were performed using R V.3.2.2 software [37].

## 3. Results

### 3.1. Within-Herd Seroprevalence and Herd-Level Prevalence

A total of 4876 blood samples from the same number of lactating cows were obtained from 150 herds. An overall individual seroprevalence of 5.3% (95% CI 2.9–7.7) was estimated; however, the within-herd seroprevalence by herd size was different. The smallest was for large herds (1.9%; 95% CI 0.1–3.8), followed by the medium-sized herds (2.8%; 95% CI 0.01–5.8). The largest was for small herds (11.6%; 95% CI 7.3–15.9), and the difference was statistically significant (*p*-value < 0.01).

The estimated overall herd-level prevalence was 42.2% (95% CI 34.2–50.2), and there was variation between different herd sizes. For large herds, it was estimated to be 28.6% (95% CI 9.2–47.9), for medium-sized herds, it was 22.3% (95% CI 7.9–37.3), and for small herds, it was 51.6% (95% CI 41.5–61.6).

It was observed that there are a more significant proportion of positive reactors in the Los Rios region (7.1%) than in the Los Lagos region (4.1%) (*p*-value < 0.05).

Non-vaccinated herds presented more significant proportions of reactors (12.2%) than vaccinated (1.1%), and the difference was statistically significant (*p*-value < 0.01).

### 3.2. Individual Prevalence by Serovars

From the serological reactors, 87.4% seroconverted to serovars included in panel I, 8.4% to those from panel II, and 4.2% co-agglutinated. The serovar Hardjo was the most seroconverted in panel I, with 46%, and serovar Tarassovi was the most seroconverted in panel II, with 7.7% (Table 1).

The serovars Autumnalis, Canicola, Pomona, and Hardjo were more reactive in the Los Rios region than in the Los Lagos region, and the difference was statistically significant (*p*-value < 0.05); and serovar Tarassovi was significantly more frequent in the Los Lagos region (*p*-value < 0.05). However, there were no statistically significant differences between the regions for Ballum, Bratislava, Grippotyphosa, and Icterohaemorrhagiae serovars (Table 2).

When evaluating the prevalence of serovars by region, herd size, and *Leptospira* vaccination history, serovars Hardjo and Pomona were more frequent in the Los Rios region, especially in small herds with no *Leptospira* vaccination history. In contrast, the serovar Tarassovi was more frequent in the Los Lagos region. In addition, these serovars were constantly observed in non-vaccinated animals.

The distribution of serovars was also different by herd size, where Hardjo and Pomona serovars were more frequent in the small herds (*p*-value < 0.01), whereas serovar Ballum was more frequent in the medium-sized herds than in small or large ones (*p*-value < 0.01). Conversely, serovars Autumnalis, Canicola, Icterohaemorrhagiae, Bratislava, and Tarassovi were observed in the same proportions in all herd sizes. In addition, it was observed that in the Los Lagos region, the proportion of reactors to serovar Canicola was more frequent in the small herds than in the medium ones (*p*-value < 0.01). Conversely, in the Los Rios region, serovars Hardjo and Pomona were more frequent in small herds (*p*-value < 0.01) and serovar Ballum was more frequent in the medium herd sizes (*p*-value < 0.01).

Animals from herds whose owners did not know the background status of pathogenic *Leptospira* spp. in their farms reacted more frequently to the serovars Canicola, Hardjo, and Tarassovi (*p*-value < 0.01). In contrast, animals from a herd with a known background of a positive diagnosis of pathogenic *Leptospira* spp. reacted more frequently to serovar Ballum (*p*-value < 0.01).

Animals from non-*leptospira*-vaccinated herds reacted more frequently to serovars Autumnalis, Pomona, Hardjo, and Tarassovi than those from *Leptospira*-vaccinated herds (*p*-value < 0.01). However, animals from the vaccinated herds reacted more frequently to the serovar Ballum (*p*-value < 0.01). In addition, serovars Canicola, Icterohaemorrhagiae, Bratislava, and Grippotyphosa were observed with the same frequency in animals from both non-vaccinated and vaccinated herds.

### 3.3. Risk Factors

#### 3.3.1. Unconditional Analysis

Table 3 shows the results obtained from the questionnaire administered to the 147 herds. A total of 27 variables were used for the analysis; of them, 13 variables were selected from the univariate analysis for further analysis: location of the farm (region), herd size, type of dairy farming (only dairy or mixed herd (dairy and beef)), heifer replacement from external sources (yes/no), cow replacement from external sources (yes/no), presence of dogs in the farm, use of a bull for mating (yes/no), use of artificial insemination (yes/no), distribution of calving (seasonal or continuous), use of vaccine against leptospirosis, background of leptospirosis, ever introduced cattle from an external source (yes/no), and regular buying of animals from external sources (open/close).

#### 3.3.2. Conditional Analysis

After evaluating interactions and potential confounding factors, Table 4 summarizes the final conditional logistic regression model for factors associated with the leptospirosis status of the farm. The final model contained three variables. All the variables included in the final model were statistically significant, and they are associated with herd management, such as the use of a bull for mating (OR = 3.43; 95% CI = 1.1–10.1) and increase in the risk of being an infected herd compared to those herds where bulls were not used for mating. In addition, herds with continuous calving along the year have a higher risk of infection (OR = 3.4; 95% CI = 1.3–8.8) than those with seasonal calving. Finally, the use of leptospirosis vaccines in the herd is a protection factor (OR = 0.04; 95% CI = 0.02–0.11).

## 4. Discussion

This study is the first stratified random survey to investigate the seroprevalence of *Leptospira* on dairy farms in Chile. On the one hand, the seroprevalence of pathogenic leptospires in lactating cows from dairies in Los Lagos and Los Rios regions was 5.3%, similar to the 4.3% reported in Spain [18] but lower to the 55.2% reported in Paraná and Sao Paulo states, in Brazil [11,15]. On the other hand, the herd-level seroprevalence was 42%, similar to the 43% reported in Spain for beef and milk herds [18]. In Brazil, larger herd prevalence levels were estimated for Paraná, Sao Paulo, Maranhao, and Paraiba states (66.6%, 70.3%, 64.8%, and 89.7%, respectively) [11,12,38]. This prevalence is lower than in other countries with more favorable conditions for the maintenance of the bacteria. Environmental factors are relevant aspects to be considered when comparing prevalence between countries as it has been shown that climatic conditions are determinants of infection [5,39], as reflected in the serological response. For example, the prevalence levels were higher in Brazilian studies, in areas with higher-temperature environmental conditions (17–27 °C) and rainfall (1300–1893 mm). These differences in the prevalence levels, both at the individual level and at the herd level, could partially be explained by the cut-off point (1:100) used to interpret MAT results in Brazil [11,12,13,15,38], which was lower than the one we used. Another aspect that could explain the difference in the prevalence levels is the number of serovars used in the MAT panel in the different studies, since there could be an increased probability of detecting infected animals at the individual and herd levels if more serovars were included within the diagnostic panel. For example, several Brazilian studies used a battery of 22 serovars [11,12,38], compared to the present one, where we used 9. Nonetheless, in our study, adding three serovars to the regular panel resulted in a 0.5% and 2% increase in the individual and herd-level prevalence, respectively.

Dairies located in Los Rios region showed the greatest proportion of reactors to pathogenic leptospires compared to those in the Los Lagos region; again, it could be due to many wetlands, which facilitates the survival of *Leptospira* in the environment. Faine et al. [40] reported that the bacteria could survive in mud, swamps, streams, and rivers. In addition, the region is extensively covered by a cold rainforest, which shelters a varied number of wild species that serve as maintenance hosts of the bacteria [41].

The most significant prevalence was observed on small farms (11.6%), which could be a consequence of the lower use of vaccines in their prevention schemes. Our results agree with those reported in the same area by [26], where only 4.3% of small dairies used vaccines against pathogenic *Leptospira* spp. However, in Chilean production conditions, smallholders also adopted fewer management practices, such as biosecurity measures and regular rodent control, increasing the risk of infection among the animals in their farms [42].

The most reactive serovar was *L. borgpetersenii* serovar Hardjo, which is not surprising as it is a common finding in other studies [6,14,16,43]. For example, Adler and de la Peña-Moctezuma [6] considered this serovar adapted to the bovine species, serving as a maintenance host for this pathogen, which explains its wide spread. In addition, *L. borgpetersenii* serovar Hardjo is not a significant cause of abortions [44]. Moreover, it appears that *L. borgpetersenii* serovar Hardjo is less pathogenic than *L. interrogans* serovar Hardjo (subtype Hardjoprajitno), which could be the reason abortions and fertility issues are more often described in the UK [45] and Australian dairies [46] and for mortality in calves [27]. Another study in the same area in Chile reported a large proportion of reactors (81%) for the serovar Hardjo as the most frequent [26]; however, the study included only small dairy farms. In addition, isolation has confirmed the presence of both serovars (Hardjobovis and Hardjoprajitno) in the area [27,28]. However, given the serovar in MAT is not capable of distinguishing among all these types, the relative frequency of each of them remains unclear. Nevertheless, our results suggest the endemic character of all Hardjo subtypes in dairy cattle in southern Chile.

*L. interrogans* serovar Pomona was the second serovar in importance. Faine et al. [40] considered serovars Pomona and Hardjo responsible for chronic disease conditions associated with fetal infections and births of premature and weak calves. *L. interrogans* serovar Pomona incidentally affects cattle, causing acute clinical cases. Faine et al. [40] reported their presence in sheep, goats, and pigs, while the results in this work found no statistical association when cattle co-grazed with these animal species. However, wildlife maintenance hosts such as rodents could carry this pathogen and explain the high frequency. Luna et al. [4] demonstrated *L. interrogans* serovar Pomona in wild rodents (*Abrothrix olivaceus*) in 64.3% of the reactors, captured from 11 dairy herds in southern Chile. It would suggest that these rodents contribute to the maintenance of this serovar in the environment, contaminating spaces where cattle pass or graze.

*L. interrogans* serovars Hardjo and Pomona were the most prevalent serovars in the present study, and both were more frequent within the Los Rios region among animals from *Leptospira* non-vaccinated farms, and both are included in commercial vaccines available in the country. However, this evidence was inconclusive that *Leptospira* vaccination practices on Chilean dairy farms were protective for *L. interrogans* serovars Hardjo and Pomona. In the present study, only a serological test was used, and, for example, testing the presence of the bacteria in urine was not included. Previous work suggests that microscopic agglutination test (MAT) results after vaccination are weak and of shorter duration, whereas titers to natural infection are stronger and persist for longer [35,47]. It is difficult to use a MAT result to distinguish between *Leptospira*-vaccinated and naturally exposed cattle because vaccinated cattle subsequently exposed to the live organism can have a strong antibody response despite being protected. In our study, 56% of the animals belonged to farms with a history of vaccination against leptospirosis; therefore, we followed OIE´s recommendations to interpret MAT in animals with a history of *Leptospira* vaccination. Thus, we used a more conservative cut-off point than other studies to reduce the effect of post-vaccination antibodies on the interpretation of the MAT results.

*L. borgpetersenii* serovar Tarassovi was another serovar with a significant distribution. Although its prevalence was low, it was present in small non-*leptospira*-vaccinated farms. In Brazil, Hashimoto et al. [15] reported it as the second in importance in the State of Paraná, and in New Zealand, Yupiana et al. [48] reported it as the third in importance. Besides, Faine et al. [40] identified *L. borgpetersenii* serovar Tarassovi as an adapted and common serovar in pigs. It agrees with the report by [49]. In this study, it was isolated from pig kidneys in slaughterhouses in Valdivia. In addition, this serovar was present in other animal species, for instance, in wild murine rodents, as reported by [41]. In New Zealand, it has been reported as an emerging serovar [7] and still poses a public health risk, especially for farmers. This serovar is not included in the commercial vaccines used in the country and is not usually included in the MAT panel; therefore, the results of this study urge us to consider this to take control measures either for animals and humans.

*L. borgpetersenii* serovar Ballum was also present in the dairies in southern Chile, with a prevalence of 0.54%, and it was more frequently in non-*leptospira*-vaccinated farms. Several studies [6,50,51] attribute the persistence of Ballum in the environment to *Mus musculus* mice, which serve as hosts and are a relevant source of infection to cattle.

Plunkett et al. [52] stated that the antibodies found in *Leptospira*-vaccinated cattle are specific for serovars included in the vaccine. Therefore, it is a reason to think that serovars Ballum and Tarassovi reported in this study could be generating infection and circulating among the vaccinated animals of these two regions since they are not included in the commercial vaccines used in the country. For this reason, it is of enormous necessity to use other diagnostic tools that allow us to know better the status of infection in the Chilean cattle population. Contrastingly, the mechanisms of antibody production generated by *Leptospira* vaccines with multiples serovars or if there is a cross response to serovars within each serogroup is not yet evident [53]. Furthermore, studies of vaccine efficacy against other serovars not included in the vaccine are rarely carried out, so their absolute protection is unknown. Therefore, annual *Leptospira* vaccinations are recommended due to the temporary and limited immunity they generate in cattle [53].

Contrasting results were reported on the relation between antibodies against MAT and elimination of the bacteria via urine. Leonard et al. [54] suggested that antibodies are related to eliminating *Leptospira* by urine in experimentally infected animals. However, [55] observed urine shedding in animals with antibodies and with titers of <1:100 identified with MAT, indicating an inverse correlation between the presence of antibodies in blood and urine. Gerritsen et al. [56] stated that the immune response does not indicate an animal is shedding the bacteria in the urine. These findings are mainly reported in a chronic state of the disease, when the bacteria reach specific target organs that allow them to be maintained for later elimination [57]. It means that seroprevalence studies are limited when detecting the true animal status, focusing solely on the determination of circulating serogroups within a population [58]. To confirm the status of the animals, the studies require confirmation employing isolates, which can be 50% different from those found by serological means [59].

On average, sera show a seropositive reaction to one serogroup with a maximum of four. Certainly, MAT is a complex test to control, perform, and interpret given the high degree of cross reaction between different serogroups, especially in acute-phase samples [60]. However, an average of one serogroup per sample was similar to other studies, suggesting a significant proportion of the reactors were in a more chronic phase or just exposed [58].

Among the potential risk factors associated with a herd with at least one positive reactor, the use of bulls was identified as a risk factor associated with herds infected with pathogenic *Leptospira* spp. Similar results were found by [11] in Sao Paulo and Laos [43]. Nevertheless, the role of the bull in disseminating the bacteria is still controversial since it has not been possible to demonstrate the presence of *Leptospira* in semen samples from animals with MAT-positive titers [61]. Furthermore, in dairy herds from southern Chile, bulls are mainly used to improve reproductive efficiency by improving heat detection and mating repeated cows; therefore, they could spread the bacteria to susceptible cows.

Another factor associated with the status of infection at the herd level was the distribution of calving, where the farms that have non-seasonal calving (continuous throughout the year) had a greater risk of having at least one reactor to pathogenic *Leptospira* spp. compared to herds with seasonal calving (spring and autumn). This higher risk could be related to a larger accumulation of water in the wintertime, contributing to greater exposure of susceptible animals during the rainy season, which coincides with the calving time. For example, a study in New Zealand demonstrated a more significant number of cases of Leptospirosis in winter and early spring, when young animals come into contact with adults disseminating infection and where humidity conditions are considerably high [62]. In addition, in Scotland, an increase in abortions caused by the serovar Hebdomadis was observed during the autumn and winter seasons (October–January), coinciding with more rainfall and environmental humidity [63]. These environmental factors may increase the risk of infection because animals have more contact with flood areas and water contaminated with urine from the host reservoirs of the infection. In the same way, a source of pollution could be represented by the rodent dejections that invade the barns during the winter, looking for heat and shelter.

The usage of vaccines against *Leptospira* (OR = 0.04) was associated with a lower risk that a herd has at least one reactor than a herd that was not vaccinate. This is in contrast to what was reported in beef cattle in New Zealand, where *Leptospira* vaccination was a predisposing risk factor to seroconvert to serovar Hardjo and Pomona [17], and another study of small herds in southern Chile [26], where the usage of vaccines against *Leptospira* increased the risk of seropositivity to *Leptospira*. However, the different criteria used to estimate the risk factor concerning the model used and the individual cut-off point of the diagnostic test to define the herd as positive are debatable.

Risk factors reported in other studies, such as co-grazing with other animal species (domestic and wild) [12,13,29,30], were statistically not significant in our study because 84% of the farmers reported that their cattle co-grazed with domestic species and 97% reported co-grazing with wild species; therefore, there was a slight chance to detect such a difference in these settings.

## 5. Conclusions

*Leptospira* are present in South Chile dairy herds and remain a risk to herd managers, their families, and employees. We estimated that in the study area, the seroprevalence of the nine pathogenic *Leptospira* spp. serovars considered was moderate to high at the herd level but low at the individual animal level in lactating dairy cows. Different frequencies of serovars are present in lactating dairy cows that are not immunized compared to those that have received immunization against *Leptospira*. Some reproductive management practices that we came across during our study were related to the chance of a herd having at least one reactor animal against pathogenic *Leptospira* spp.

## Figures and Tables

**Table 1:** Proportion of animals reacting to each serovar included in the MAT panel and prevalence among the dairy cows of southern Chile.

Panel	Serovar	Seropositive		Prevalence	
		(*n*)	(%)	(%)	95% CI
1	A	7	2.4	0.14	0.01–0.30
1	B	26	9.1	0.53	0.30–0.80
1	C	11	3.8	0.23	0.10–0.40
1	H	132	46.1	2.71	2.30–3.20
1	I	1	0.3	0.02	0.01–0.10
1	P	73	26.0	1.50	1.20–1.90
2	Br	1	0.3	0.02	0.01–0.10
2	G	1	0.3	0.02	0.01–0.10
2	T	22	7.7	0.45	0.30–0.70
	Coag.	12	4.2	0.25	0.10–0.40
	Total	286		5.87	4.9–6.8

A: Autumnalis; B: Ballum; Br: Bratislava; C: Canicola; G: Grippotyphosa; H: Hardjo; I: Icterohaemorrhagiae; P: Pomona; T: Tarassovi; Coag: Co-agglutination.

**Table 2:** Frequency of each serovar included in the MAT panel by herd location, herd size, and *Leptospira* vaccination status of the herd (cut-off titer for non-vaccinated ≥1:200 and for vaccinated ≥1:800) among the lactating dairy cows of southern Chile (*n* = 4768).

Panel	Serovar	Herd Location (*n*)		Herd Size (*n*)			Vaccination Status (*n*)	
		Los Lagos (1691)	Los Ríos (3077)	Large (1054)	Medium (1473)	Small (2241)	Non-Vaccinated (2119)	Vaccinated (2649)
1	A	0	7 *	0	1	6	7	0
1	B	6	20	6	1	19 **	17	9 *
1	C	1	10 *	1	2	8	10	1 **
1	H	21	111 *	6	23	103 **	128	4 **
1	I	0	1	0	0	1	1	0
1	P	12	61 *	9	8	56 **	67	6 **
2	Br	0	1	0	1	0	1	9
2	G	1	0	0	1	0	1	0
2	T	17 *	5	2	8	12	17	5 **
	Coag.	11	1 **	1	9	2 **	10	2
	Total	69	217	25	54	207 **	259	27 **

A: Autumnalis; B: Ballum; Br: Bratislava; C: Canicola; G: Grippotyphosa; H: Hardjo; I: Icterohaemorrhagiae; P: Pomona; T: Tarassovi; Coag: Co-agglutination. * *p* < 0.05; ** *p* < 0.01.

**Table 3:** Prevalence of and risk factors for leptospirosis in South Chile dairy herds.

Herd-Level Characteristic	Category	Herd Leptospirosis Status	
		**Pos.**	**Neg.**
Location of the farm (region)	Los Ríos	48	56
	Los Lagos	14	29
Herd size	Small	49	46
	Medium	7	24
	Large	6	15
Type of dairy farming	Dairy only	48	75
	Mixed herd	14	10
Heifer replacement from external sources	Yes	14	9
	No	48	76
Cow replacement from external sources	Yes	48	76
	No	14	9
Presence of dogs in the farm	Yes	48	73
	No	14	12
Use of a bull for mating	Yes	52	52
	No	10	33
Use of artificial insemination	Yes	33	55
	No	29	30
Distribution of calving	Seasonal	29	52
	Continuous	33	33
Use of vaccine against leptospirosis	Yes	6	61
	No	56	24
Background of leptospirosis	Yes	4	18
	No	58	67
Ever introduced cattle from an external source	Yes	22	20
	No	40	65
Regular buying of animals from external sources	Open	41	40
	Close	21	45
Regular application of rodent control measures	Yes	43	62
	No	18	22
Regular rodent outbreaks on the farm	Yes	33	51
	No	29	34
Regular application of ectoparasites control measures	Yes	60	85
	No	2	0
Animals in contact with neighboring animals via fences	Yes	37	48
	No	25	37
Disposal of carcasses within the farm	Yes	61	82
	No	1	3
Use of feeding yard for heifers	Yes	59	79
	No	3	4
Use of feeding yard for cows	Yes	55	70
	No	7	15
Type of milking	Manual	8	13
	milking machine	54	72
Calf-raising system	with the dam	22	24
	Artificial rearing conditions	40	61
Bovines sharing paddocks with other domestic animals (pigs, sheep, horses)	Yes	8	14
	No	54	71
Grouping of cows based on productivity	Yes	11	12
	No	51	73
Paddocks only for heifers	Yes		
	No		
The herd size of the farm (in the last 5 years)	Increased	34	37
	Remained stable	23	35
	Decreased	5	13
Presence of wildlife on the farm	Yes	59	83
	No	2	2

**Table 4:** Conditional logistic regression model results showing the factors associated with infection by pathogenic *Leptospira* spp. at the herd level using MAT in herds of southern Chile.

Variable	Category	OR	95% CI	*p*-Value
Use of a bull for mating	No	Ref.		
	Yes	3.43	1.16–10.14	0.026
Distribution of calving	Seasonal	Ref.		
	Continuous	3.38	1.30–8.79	0.012
Use of vaccine	No	Ref.		
	Yes	0.04	0.02–0.11	<0.01

## Data Availability

The data presented in this study are available on request from the corresponding author. However, the data are not publicly available due to the confidentially of the farmers’ information.

## Supplementary Materials

The following are available online at https://www.mdpi.com/article/10.3390/ani11113148/s1, Questionnaire.
