# Supplementary material for: Pathogenic Leptospira spp. Seroprevalence and Herd-Level Risk Factors Associated with Chilean Dairy Cattle
Source: Animals (Basel). 2021 Nov 4;11(11):3148. doi: 10.3390/ani11113148 (PMC8614305; doi:10.3390/ani11113148)
Supplement: Supplementary file 1 [file animals-11-03148-s001.zip › animals-1391100-supplementary.pdf]

ENCUESTA PREDIAL PROYECTO FONDECYT, ***PRODUCTORES***

EPIDEMIOLOGÍA

PREVALENCIA PREDIAL Y VALIDACIÓN DE TRES TÉCNICAS DIAGNÓSTICAS PARA TRES ENFERMEDADES IMPORTANTES EN LECHERÍAS DEL SUR DE CHILE

OBJETIVO: Identificar factores de riesgo prediales relacionados con la tasa de infección predial

ENCUESTADOR:______________________________________ Codigo_____________Latitud:__________Long: ___

Nombre:_________________________________ Fecha:_________ Comuna______________________ NºSAG_____

Propietario:________________________________ N teléfono:___________________ ____________________

Referencia para llegar a lechería. Camino (de a)__________________________________ Desde__________ Km_______

Ubicación lechería:_______________________________________ Jefe lechería:________________________________

Med. Veterinario:__________________________ Teléfono:______________ ______________ N visitas al mes:____

1 INFORMACION PREDIO: **Codigo**__________

1.1 **Superficie destinada a vacas lechería:** __________Has

Clasificación del tipo de explotación: Lechería __si __no Carne __si __no Agricultura __si __no

2 DATOS DEMOGRAFICOS

2.1 **Raza predominante vacas:** ____

2.2 **Cantidad de animales:**

**Hembras bovinas**	**N°**	**Machos bovinos**	**N°**
Terneras (<1año)		Terneros (<1año)	
Vaquillas vírgenes		Novillos	
Vaquillas encaste		Toros	
Vaquillas preñadas		Bueyes	
Vacas masa			

2.3 En los últimos 5 años su predio: a) creció en el número de animales___ b) se mantuvo estable:____ c) Se redujo___
En cuántos?_____

3 REPOSICIÓN DE ANIMALES

3.1 **Origen de la reposición de vaquillas/vacas:** Propia __si __no Compra __si __no

3.1.1 Elegir ¿**Cuando compra**? __Siempre __A veces

3.1.2 ¿**Donde compra**? __Feria __Otro productor __Criadero con pedigrí __Remate especial Otra parte____________

3.1.3 ¿**De que tipo de predios provienen las vaquillas**?

Libres de: Leptospirosis __Si __No Paratuberculosis bovina: __Si __No Diarrea viral bovina:__Si __No

3.1.4 ¿**Solicita certificado sanitario de los animales comprados**? (que sean negativos a alguna prueba diagnóstica)

Libres de: Leptospirosis:__Si __No Paratuberculosis bovina: __Si __No Diarrea viral bovina:__Si __No

3.1.5 ¿**Que otras categorías de animales se compran?** ____

3.1.6 ¿**Dónde se compran estos animales**?____

3.1.7 **SI NO COMPRA**; las vaquillas o terneras se crian aparte? __si __no. Se juntan de varias lecherías? __si __no

4 ALIMENTACION

4.1 **De las vaquillas últimos 6 meses de edad:**

4.1.1 Sólo pradera : Si__ No__ Comparten bebederos con otro grupo? Si__ No__ Cuál?_____

4.1.2 Suplementación mineral: Si__ No__

4.2 **De las vacas en producción:**

4.2.1 ¿Se separan las vacas por lotes de alimentación de acuerdo a producción? Si__ No__

4.2.2 Sólo pradera : Si__ No__ Comparten bebederos con otro grupo? Si__ No__ Cuál?_____

4.2.3 Suplementación mineral: Si__ No__

5 ANTECEDENTES PASTOREO GENERAL

5.1 Presencia de perros Si__ No__

5.2 Los bovinos comparten la pradera con ovinos Si__ No__ caballos Si__ No__ cerdos Si__ No__
otro animal_________________ Y bebederos? Si__ No__. Si es sí: con qué especies?:__________

5.3 Presencia visible animales silvestres en el predio Si__ No__. Cuales __zorro __puma __ pudú __jabalí
Liebres____ Conejos ____ Otro_______

6 INSTALACIONES Y MANEJO TERNEROS (predestete)

6.1 **Crianza:** __Natural __Artificial:

6.1.1 ¿Cuantos días permanece con la madre después del parto? ____ horas/días

6.2 ¿**Existe contacto de los terneros con sus madres después de 24 h nacido**? Si__ No__ .

6.3 Se lavan las ubres o pezones antes de colectar el calostro o dar de mamar a los terneros? Si__ No__

6.4 **Ternereras:** __ Individuales __Colectivas

6.4.1 Tienen acceso a corral (sin pasto) Si__ No__

6.4.2 Tienen acceso a potrero (con pasto) Si__ No__

6.5 Desinfecta las ternereras luego de la salida de los animales?

6.6 ¿**Los terneros tienen contacto con bovinos mayores de un año**? Si__ No__

6.7 ¿Los **terneros tienen contacto con otras especies de animales, es decir, comparten praderas o corrales o galpón**?
Si__ No__ Comparten bebederos con otro grupo? Si__ No__ Cuál?_____

6.8 ¿**Los potreros de los terneros son de uso EXCLUSIVO de ellos** (sin rotación con otros de mayor edad)? Si__ No__

7 INSTALACIONES Y MANEJO RECRIA (DESDE DESTETE A SERVICIO)

7.1 Se utiliza: Potrero permanente (sin encierro) __Si __No Todo el año? __Si __No Cuando encierran?_________
7.2 Encierro nocturno__Si __No (__Galpón, __Potrero)
7.3 Patio de alimentación __No __todo el año __estacional

7.4 ¿**Los terneros son separados por sexo?** Si__ No__, en que momento los separa?_______________________
7.5 ¿**Los terneros ya destetados vuelven alguna vez a las ternereras**? Si__ No__
7.6 ¿Por qué causas?:____

7.7 Tipo del contacto con animales mayores de 1 año
7.7.1 Potreros vecinos__Si __No
7.7.2 Rotación potrero__Si __No
7.7.3 Pastoreo conjunto__Si __No
7.8 Vaquillas al encaste: Peso_________Kg Edad___meses

8 INSTALACIONES Y MANEJO VACAS EN PRODUCCION
8.1 Se utiliza: Potrero permanente (sin encierro) __Si __No Todo el año? __Si __No Cuando encierran?_________
8.2 Encierro nocturno__Si (__Galpón, __Potrero) __No
8.3 Patio de alimentación __No __todo el año __estacional

9 ORDEÑA
9.1 Tipo de ordeña: __manual __mecánica
9.2 Número de unidades de ordeña___
9.3 Número de ordeñas__ al día
9.4 Tipo de disposición unidades: __Espina pescado __ Tandem __Salida frontal __Rotatoria

9.5 **Causas por las que descarta la leche**: ____
9.6 **Si descarta calostro**, cómo lo hace:
__Terneros fresco __Terneros acidificado __Terneros calentada
__a otros animales, cuales ____
__Eliminación, dónde____
9.7 **Destino leche de la otra leche de descarte** (elegir):
__Terneros cruda __Terneros acidificada __Terneros calentada
__a otros animales, cuales ____
__Eliminación, dónde__________________
9.8 Destino de efluentes lechería:
9.8.1 Del agua de lavado del equipo de ordeña___________________________
9.8.2 De los purines y agua de lavado de la sala___________________________

10 INSTALACIONES Y MANEJO PARA VACAS SECAS (preñadas y fuera de ordeña)
10.1 Se utiliza: Potrero permanente (sin encierro) __Si __No Todo el año? __Si __No Cuando encierran?_________
10.2 utiliza lugares exclusivamente para las pariciones? __Si __No.
10.3 Patio de alimentación (elegir) __No __todo el año __estacional

11 SISTEMA DE CRUZAMIENTO
11.1 **Uso de toro**: __Si __No Procedencia:______________________ Cuanto tiempo que lo tiene o rota:___________
11.2 **Para qué animales utiliza toro** (elegir) __vacas __vaquillas __sólo para repaso luego de _________repeticiones de IA
11.3 ¿Usa toro celador para detección de vacas o vaquillas en celo? __Si __No
11.4 Uso de Inseminación Artificial__Si __No Proveedor:___________________
11.5 Tipo de IA (elegir): __predial __posta móvil Toros Nacionales: _______ Importados:__________
11.6 Para qué animales utiliza IA: Vacas __Si __No (elegir): __Todas __Algunas
Vaquillas __Si __No (elegir): __Todas __Algunas
11.7 Cómo es la distribución de los partos __Continua en el año __Estacional:
__primavera__% __otoño__%
11.8 ¿**Realiza revisión posparto en las vacas**? __Si __No

12 MANEJO SANITARIO PREDIAL
12.1 Vacunaciones realizadas en el predio el último año:
__IBR __DVB __Leptospira __Brucelosis Combinadas:________ o solas:_________________
12.2 Condición del predio (Elegir: 1.libre, 2 en saneamiento, 3 no controla) para:
Brucelosis______ Tuberculosis_____ Leptospirosis_____ Leucosis:______

13 BIOSEGURIDAD AMBIENTAL

13.1 Disposición de cadáveres en el predio.

__ Entierro ___ Quema ___ No realiza Otro:________________________

13.2 Sus animales tienen contacto directo con animales de sus vecinos a través de cercos ? Si _ No _

13.3 Esparcen los purines en las praderas? Si _ No _ Con qué frecuencia?_____ Qué dosis?________

13.4 Tienen problemas con roedores?______

13.5 Control de roedores (marcar con una X)

Control de roedores con rodenticida			
	Si	No	Frecuencia
Ternereras			
Lechería			
Galpón alimentos			

14 Control Ectoparásitos . Si _ No _

14.1 ¿Cómo lo realiza? ____

14.2 ¿Con qué frecuencia? ____

14.3 ¿Hay en el establecimiento? tábanos? Si _ No _ Zancudos? Si _ No _ Mosca de los Cuernos? Si _ No
Mosca establo? Si _ No _

15 Vacunación:

15.1.1 ¿Quién realiza: * vacunaciónes ____.

15.2 Vacunación brucelosis? ____.

15.3 Sanidad? ____.

Ud. Conoce la enfermedad paratuberculosis? Hizo alguna vez algún análisis Si _ No cuál fue su resultado?

Ud. Conoce la enfermedad leptospirosis? Hizo alguna vez algún análisis Si _ No cuál fue su resultado?

Ud. Conoce la enfermedad Diarrea viral bovina? Hizo alguna vez algún análisis Si _ No cuál fue su resultado? ___

En qué categoría de animales los hicieron (edad)?____________

Tiene problemas de terneros débiles, que no coordinan bien?____________

Abortos?__________________ A donde los enviaron?_________________

Gracias pos su colaboración.

16 OBSERVACIONES.

____
____
____
____
____
____
____
____
____
____
